# From Angiosome to Woundosome: An Interdisciplinary Approach to Personalized Revascularization in Chronic Limb-Threatening Ischemia

**DOI:** 10.3390/diagnostics16101557

**Published:** 2026-05-20

**Authors:** Mircea Ionut Popitiu, Lorenzo Patrone, Giacomo Clerici, Serban Comsa, Gloria Gavrila-Ardelean, Nilima Rajpal Kundnani, Nicu Olariu, Mihai Edmond Ionac

**Affiliations:** 1 Research Center in Vascular and Endovascular Surgery, “Victor Babes” University of Medicine and Pharmacy, 300041 Timisoara, Romania; mihai.ionac@umft.ro; 2 Vascular and Endovascular Surgery Unit, San Giovanni di Dio Hospital, 50143 Florence, Italy; patropatronis@gmail.com; 3 San Carlo Clinic, 20026 Paderno Dugnano, Italy; gclerici@casacura.it; 4 Angiogenesis Research Center, Department II Morphologic Microscopy/Histology, “Victor Babes” University of Medicine and Pharmacy, 300041 Timisoara, Romania; serban.comsa@umft.ro; 5 Faculty of Educational Sciences, Psychology and Social Work, ”Aurel Vlaicu” University of Arad, 310032 Arad, Romania; 6 University Clinic of Internal Medicine and Ambulatory Care, Prevention and Cardiovascular Recovery, Department VI-Cardiology, “Victor Babes” University of Medicine and Pharmacy, 300041 Timisoara, Romania; knilima@umft.ro; 7 Research Center of Timisoara, Institute of Cardiovascular Diseases, “Victor Babes” University of Medicine and Pharmacy, 300041 Timisoara, Romania; 8 Division of Nephrology, Department of Internal Medicine II, “Victor Babes” University of Medicine and Pharmacy, 300041 Timisoara, Romania; nicu.olariu@umft.ro; 9 Center for Molecular Research in Nephrology and Vascular Disease, “Victor Babes” University of Medicine and Pharmacy, 300041 Timisoara, Romania; 10 Doctoral School, “Victor Babes” University of Medicine and Pharmacy, 300041 Timisoara, Romania

**Keywords:** chronic limb-threatening ischemia, angiosome, woundosome, revascularization, diabetic foot, tissue perfusion, collateral circulation, endovascular therapy, clinical decision-making

## Abstract

**Background/Objectives**: Chronic limb-threatening ischemia (CLTI) is the most advanced stage of peripheral arterial disease and is associated with high rates of major amputation and mortality. The angiosome concept has become an important tool for planning targeted revascularization. However, its clinical value may be limited in patients with complex arterial disease, impaired collateral circulation, and microvascular dysfunction. This review explores the relationship between angiosome-guided revascularization and the emerging woundosome concept, which focuses on functional wound perfusion. **Methods:** A narrative review with a structured literature search was performed using PubMed/MEDLINE, Scopus, and Web of Science. Studies evaluating angiosome-guided revascularization, direct versus indirect revascularization, collateral circulation, pedal arch integrity, and perfusion-related outcomes in CLTI and diabetic foot disease were included. **Results**: Most observational studies and meta-analyses suggest that direct angiosome-targeted revascularization may improve wound healing and limb salvage in selected patients. However, clinical outcomes are also influenced by collateral circulation, anatomical variability, infra-malleolar perfusion, pedal arch integrity, and microvascular function. The woundosome concept expands the traditional angiosome model by emphasizing effective perfusion of the wound bed through direct arterial inflow, collateral pathways, and functional perfusion assessment. **Conclusions**: Combining the angiosome and woundosome concepts may provide a more practical and individualized approach to revascularization planning in CLTI by integrating anatomical vascular mapping with functional wound perfusion assessment.

## 1. Introduction

Chronic limb-threatening ischemia (CLTI) is the most advanced stage of peripheral arterial disease. It is defined by severe symptoms: ischemic rest pain, ulcers that do not heal, or gangrene [1,2,3,4]. For patients with CLTI, morbidity and mortality are high; therefore rapid diagnosis and efficient revascularization are required, as highlighted in all the major international guidelines and consensus documents [5,6,7,8,9,10].

Despite advances in both surgical and endovascular interventions, clinical outcomes for patients with CLTI remain far from ideal, with high rates of persistent, non-healing wounds and, ultimately, major limb amputations [8,9,10,11]. The angiosome concept, first described by Taylor and Palmer in 1987, has become of rising importance in the revascularization treatment of patients with diabetic foot ulcers and CLTI, but has not shown consistency in terms of clinical results [2,12,13,14]. This discrepancy suggests that a purely anatomical restoration of blood flow may not be enough to solve the problem of inadequate tissue perfusion in ischemic limbs [7,15]. The blood supply of the wound bed is affected by a complex network of factors: collateral circulation, microvascular dysfunction, and interindividual anatomical variability [16,17,18]. These limits point to the strong need for complementary medical approaches that go beyond rigid anatomical models [15,19]. This review therefore aims to explore the evolving relationship between traditional angiosome-based revascularization and the emerging woundosome concept, which emphasizes functional wound perfusion.

## 2. Methods

This study is a narrative review with a structured thematic synthesis approach. The research method was adopted to allow the inclusion of diverse study designs, including observational studies, clinical cohorts, and conceptual analyses, in order to provide a comprehensive and clinically relevant synthesis.

This review was conducted within the context of contemporary vascular surgery practice, focusing on clinical decision-making in patients with CLTI and with diabetic foot disease where optimizing wound perfusion remains a major challenge. As this study is a narrative review based on previously published data, no ethical approval or informed consent was required.

This research aims to synthesize current evidence regarding the angiosome concept, angiosome-guided revascularization and emerging perfusion-oriented paradigms, including the woundosome concept, in patients with CLTI and diabetic foot disease. Given the heterogeneity of the included studies and the conceptual nature of the angiosome and woundosome paradigms, a qualitative integrative approach was considered appropriate to explore patterns, relationships, and emerging concepts across literature.

A comprehensive literature search was carried out across the electronic databases: PubMed/MEDLINE, Scopus and Web of Science. The search covered publications from the first 1987 description of the angiosome concept by Taylor and Palmer through to January 2026.

The search utilized a strategic combination of keywords to capture all relevant data. The keywords were: “woundosome”, “indirect revascularization”, “chronic limb-threatening ischemia”, “angiosome”, “direct revascularization”, “critical limb ischemia”, “diabetic foot”, “infrapopliteal intervention”, and “perfusion imaging”. Boolean operators (AND/OR) were used to ensure specific, relevant results.

Criteria for inclusion and exclusion were selected in accordance with academic standards.

Studies qualified for inclusion if they satisfied the following criteria:

1. clinical studies, observational cohort studies, randomized controlled trials, systematic reviews, or meta-analyses;
2. investigations that involve patients with CLTI or diabetic foot ulcers;
3. studies that evaluate angiosome-guided revascularization, direct versus indirect revascularization strategies or perfusion-related outcomes;
4. publications written in English.

The exclusion criteria were:

1. editorials, expert opinions without original data and small case reports;
2. studies that were not directly related to lower limb revascularization or wound perfusion;
3. duplicate publications or studies with insufficient methodological detail.

To ensure no relevant data was missed, additional pertinent studies were manually retrieved from reference lists of key articles and international guideline documents.

The selection process was non-blinded and resulted in the inclusion of 52 studies in the qualitative synthesis. A quantitative meta-analysis was not performed due to the heterogeneity in study designs, patient populations and outcome definitions.

The selected studies were analyzed qualitatively using a thematic narrative synthesis approach. Studies were stratified by key domains, including the anatomical basis of the angiosome model, clinical outcomes of angiosome-guided revascularization, the role of collateral circulation and microvascular perfusion, and emerging concepts such as the woundosome paradigm.

Comparative analysis was performed to identify recurring patterns, consistencies, and discrepancies across studies, with particular attention to factors influencing wound healing and tissue perfusion in chronic limb-threatening ischemia.

The authors have clinical experience in vascular surgery and the management of CLTI and diabetic foot disease. This clinical background may influence the interpretation of the literature toward clinically applicable frameworks. Efforts were made to provide a balanced and comprehensive synthesis of the available evidence.

To enhance the credibility of the synthesis, evidence was systematically compared across different study designs, including observational studies, clinical cohorts, and meta-analyses. Consistency and convergence of findings were used to support the robustness of interpretations, while discrepancies between studies were critically evaluated. Since this is a narrative review, formal systematic review protocols and meta-analytic methods were not applied.

This article was designed as a narrative review supported by a structured literature search and thematic synthesis rather than as a formal systematic review. This approach was chosen because the available literature on angiosome-guided and woundosome-oriented revascularization is highly heterogeneous with regard to study design, patient populations, anatomical complexity, revascularization strategies, perfusion assessment techniques, and reported outcomes. Furthermore, the woundosome concept remains an evolving clinical and physiological framework rather than a standardized therapeutic approach, making formal quantitative synthesis difficult. Therefore, a narrative review was considered more appropriate for integrating the available anatomical, hemodynamic, imaging, and clinical evidence into a practical and clinically relevant perspective.

**Figure 1:**
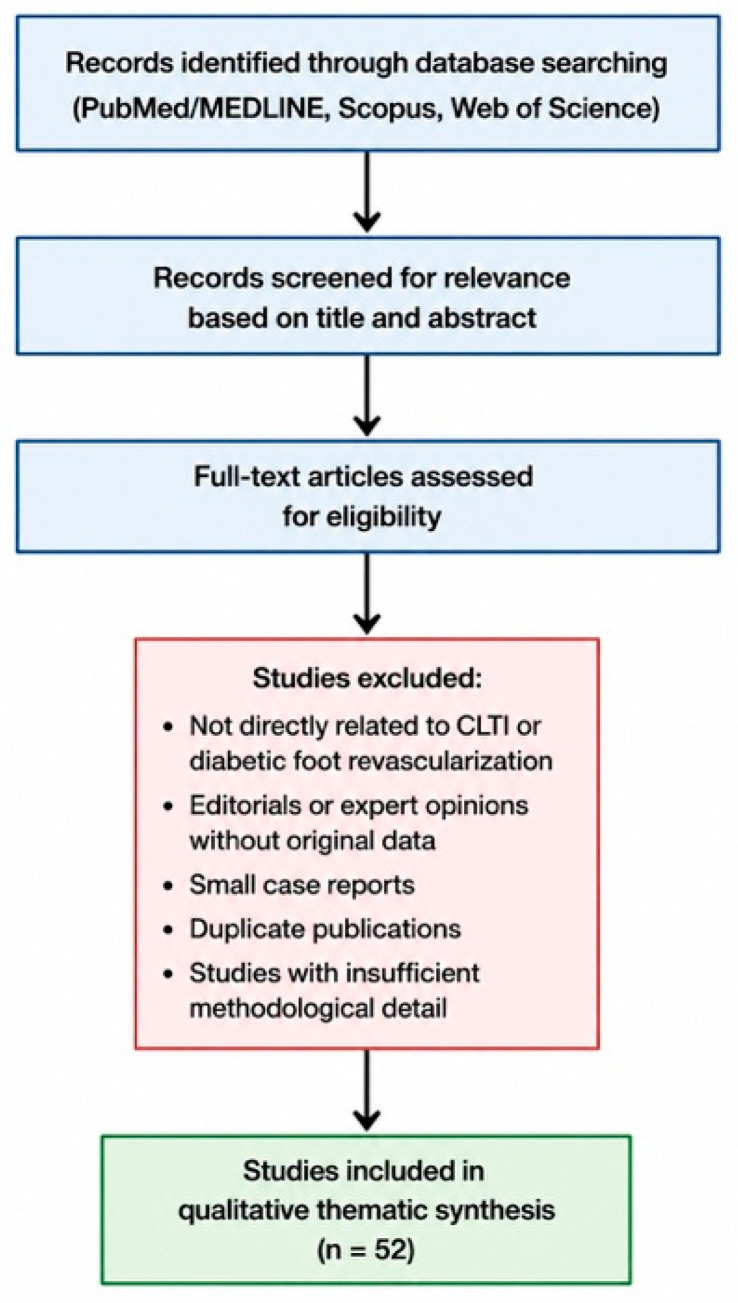
Flowchart of study selection. Literature records were identified through searches of PubMed/MEDLINE, Scopus, and Web of Science. After removal of duplicate and non-relevant articles, full-text studies were screened according to predefined inclusion and exclusion criteria. A total of 52 studies were ultimately included in the qualitative thematic synthesis.

The study selection process is summarized in Figure 1.

## 3. Results

### 3.1. Angiosome Anatomy of the Foot

The angiosome concept was pioneered by Taylor and Palmer, who redefined vascular anatomy by partitioning the body into three-dimensional units [2]. Each tissue unit is supplied by a source artery. In the lower limb, the foot and ankle are subdivided into six distinct angiosomes. These territories are supplied by the three primary infrapopliteal arteries: the posterior tibial artery (PTA), the anterior tibial artery (ATA) and the peroneal artery [2,3].

The PTA supplies three angiosomes: the medial plantar angiosome, which perfuses the medial plantar instep; the lateral plantar angiosome, which supplies most of the plantar forefoot and midfoot; and the calcaneal angiosome, which provides blood to the plantar heel through medial calcaneal branches [2,3,20]. These regions clinically correspond to many plantar ulcer locations in the diabetic foot [16,19,21].

The ATA, continuing distally as the dorsalis pedis artery, supplies the dorsal angiosome, which perfuses the top of the foot and toes. This area is especially important for dorsal foot wounds and ischemic lesions affecting the digital arteries [2,3,20].

The peroneal artery contributes to two angiosomes: the anterolateral ankle angiosome, supplied by its perforating branch, and the lateral calcaneal angiosome, which vascularizes the lateral heel and posterior-lateral hindfoot [2,20,22].

Although these angiosomes represent anatomically distinct vascular territories, they are interlinked by a network of arterial–arterial connections and “choke vessels”, which can provide collateral perfusion between adjacent territories. Vessels with a diameter of ≥1 mm are generally considered true arterial connections capable of sustaining effective collateral flow, a concept aligned with Taylor’s description of the “functional angiosome” [23]. The functional significance of these connections is especially important in patients with CLTI, where collateral circulation may affect the success of direct revascularization (DR) or indirect revascularization (IR) strategies [17,24].

Table 1 presents the angiosomes for the foot and ankle regions, the supplying arteries, and the clinical relevance. Knowledge of these vascular territories is important for planning angiosome-guided revascularization in CLTI and diabetic foot ulcer (DFU) [2,3,20].

### 3.2. The Woundosome Concept and Its Implications for Revascularization

While the angiosome model provides an anatomical framework for understanding tissue perfusion, clinical experience has shown that wound healing does not always strictly follow predefined angiosomal territories. In some cases, wounds located within a given angiosome may heal despite IR, suggesting that functional perfusion may extend beyond anatomical boundaries through collateral networks [23,25,26].

Several aspects of the angiosome concept are well established. The vascular territories of the foot, which are vascularized by the posterior tibial artery, anterior tibial artery, and peroneal artery, have been anatomically described, and the presence of arterial–arterial connections and “choke vessels” between adjacent angiosomes is well documented [2,8,20,23,24]. DR based on the angiosome concept was shown to be able to improve wound healing in selected CLTI patients, although results remain heterogeneous [5,6,7,9,10,15,19,27,28,29,30].

At the physiological level, it is plausible that wound perfusion is determined not only by the primary source artery but also by collateral circulation, microvascular integrity, and local tissue demand, mainly related to the size of the ulcer. Diabetic microangiopathy, local infection, inflammation, and significant tissue necrosis can influence perfusion patterns and the oxygenation of the wound bed [1,6,10,14,16,18,19,21,31,32,33,34,35].

Given this context, recent studies proposed a new term, “woundosome”, which refers to a functional vascular territory that is centered on the wound, not on anatomical angiosomes [19,36]. This concept supports the idea that the effective wound bed perfusion may rely on the contribution of multiple arterial pathways and microcirculatory networks, even outside of strict angiosomal classification, as in the case of anatomical variants. In addition, it states how the direct in line flow pathway to the wound, with a particular focus on the often-neglected infra-malleolar vessels, could lead to better wound-healing [19,30,36].

#### Clinical Definition of the Woundosome

The “woundosome” concept has emerged as a functional extension of the classical angiosome paradigm, shifting the focus from fixed anatomical vascular territories toward effective wound-centered perfusion [19]. The woundosome can be understood as the functional perfusion territory that maintains the wound bed through the combined effect of direct arterial inflow, collateral circulation, infra-malleolar vascular networks, and microvascular integrity. In contrast to the classical angiosome model, which is based on predefined anatomical territories, the woundosome concept focuses on the actual tissue perfusion surrounding the wound and recognizes that this may vary between patients depending on collateral circulation, distal vascular networks, and tissue condition [19,36].

In clinical practice, evaluation of the woundosome is based on correlation between wound location, angiographic assessment of arterial inflow and collateral pathways, and, when available, functional perfusion imaging techniques [15,19,30,36]. This approach may support a more individualized assessment of tissue perfusion and target vessel selection, particularly in patients in whom direct revascularization is not technically feasible or collateral circulation plays a major role [15,19,28].

**Figure 2:**
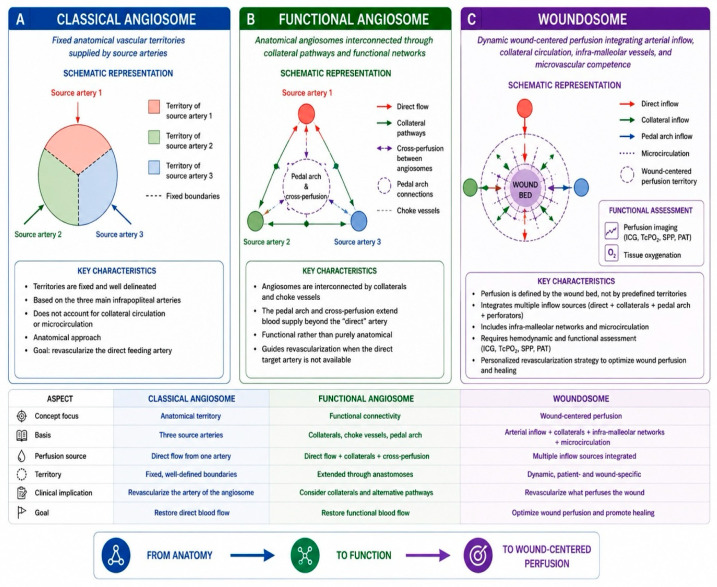
Evolution from the anatomical angiosome model to the woundosome concept. (**A**) Classical angiosome: fixed anatomical vascular territories supplied by source arteries. (**B**) Functional angiosome: anatomical angiosomes interconnected through collateral circulation, choke vessels, and pedal arch networks, allowing cross-perfusion between adjacent territories. (**C**) Woundosome: a dynamic wound-centered perfusion concept integrating direct arterial inflow, collateral pathways, infra-malleolar networks, and microvascular competence to support personalized revascularization strategies and optimize wound healing.

Rather than replacing the angiosome concept, the woundosome should be viewed as its functional and clinically oriented extension. However, it currently remains mainly a conceptual framework rather than a fully validated therapeutic model. Although advances in perfusion imaging and microcirculatory assessment may improve characterization of wound-specific perfusion patterns, further clinical evidence is still required before woundosome-guided revascularization strategies can be routinely standardized in clinical practice. The transition from anatomical angiosome-guided perfusion toward the functional woundosome concept is presented in Figure 2.

**Figure 3:**
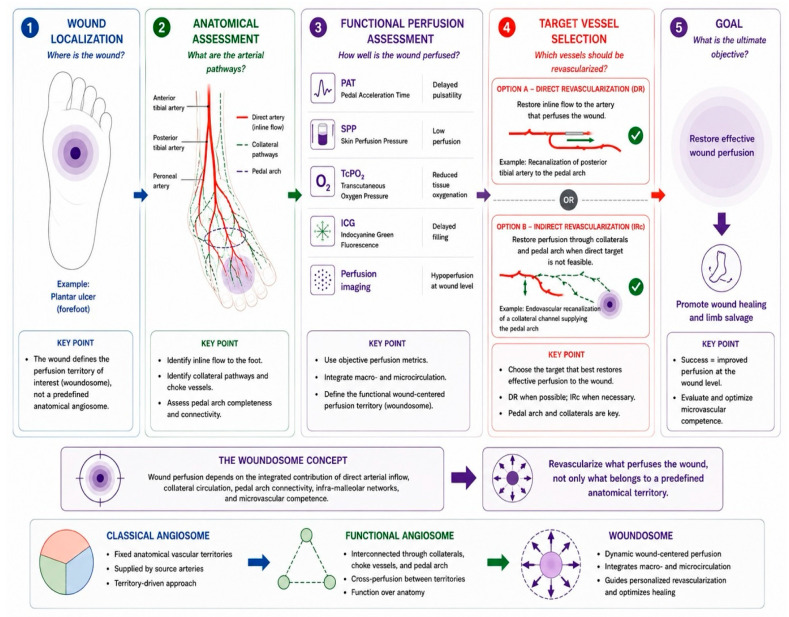
Illustrative clinical application of the woundosome concept in chronic limb-threatening ischemia (CLTI). The schematic workflow illustrates a wound-centered approach to revascularization planning based on wound localization, vascular assessment, collateral circulation, pedal arch evaluation, and multimodal perfusion assessment using pedal acceleration time (PAT), skin perfusion pressure (SPP), transcutaneous oxygen pressure (TcPO_2_), and indocyanine green (ICG) fluorescence imaging. Final target vessel selection is guided by restoration of effective wound perfusion and wound healing potential.

A schematic illustration of the clinical application of the woundosome concept in revascularization planning is presented in Figure 3.

Therefore, the woundosome concept may be best viewed as an upgraded version of the anatomical angiosome model, without rejecting its basic anatomical guidance, while highlighting the importance of functional perfusion and collateral circulation when planning revascularization strategies in patients with CLTI [37].

Recent studies explore the relationship between angiosome anatomy, collateral circulation, and functional wound perfusion. Recent literature highlights that wound perfusion may originate from multiple arterial territories through collateral circulation. These findings support the woundosome framework, which complements the angiosome model by emphasizing restoration of effective perfusion to the wound bed. The most recent key studies supporting the woundosome framework, published over the past 5 years, are summarized in Table 2, including study design, patient population, and main findings [5,16,19,20,36,38,39,40].

### 3.3. Direct Versus Indirect Revascularization

Angiosome-guided DR targets the artery supplying the ischemic angiosome containing the wound [15,18]. In contrast, IR restores blood flow through adjacent vascular territories and relies on collateral circulation to perfuse the affected tissue [19,30]. The relative effectiveness of these strategies remains an area of active investigation [28,37].

#### Clinical Evidence

Literature has been increasingly investigating the clinical effects of angiosome-guided revascularization in patients with CLTI and DFU [27,33]. The main objective of angiosome-guided DR is to bring perfusion back to the specific ischemic territory directly through its corresponding artery, for wound healing. Conversely, indirect methods, which have been studied comparatively through observational studies, are used when the affected tissue relies primarily on collateral circulation [11,27,33].

Early clinical data strongly favored the direct approach. For instance, a retrospective study by Iida et al. showed that endovascular therapy targeting the specific wound-related artery achieved significantly higher healing rates than indirect strategies [4]. Similarly, Azuma and colleagues noted that bypass surgery directed at the angiosome led to better ulcer closure and improved limb salvage in diabetic cohorts [41]. The speed of recovery also seems to favor direct revascularization. Kret et al. and Acín et al. demonstrated a shorter time-to-closure and faster overall healing of chronic ulcers after direct revascularization [29,42].

Subsequent studies have further explored the clinical relevance of the angiosome concept in the endovascular era [1]. Targeting the direct angiosome pathway yielded superior results for the resolution of DFU, as shown by Meloni et al. [14]. Hou et al. also reported favorable wound healing rates achieved through angiosome DR [21].

Several studies have evaluated revascularization based on the angiosome model in patients with CLTI. Table 3 summarizes the main characteristics and findings of the profile studies published during the past 5 years [5,14,19,21,30]. Studies conducted over a decade ago suggested that DR may be associated with improved wound healing and limb salvage compared with IR [43,44]. Recent meta-analyses, such as the 2026 study by Tarricone et al., have confirmed the benefit of angiosome-based DR for wound closure, limb salvage and survival [5].

Despite these findings, not all studies have demonstrated a clear clinical advantage of direct revascularization. Several studies have found that DR and IR can yield comparable results, particularly in patients with a well-developed collateral network [27]. In these patients, IR through neighbouring angiosomes may provide enough blood flow through collaterals to the ischemic areas, thus lowering the importance of strict angiosome targeting. These variations show that, in addition to the angiosome anatomical vascular map, functional tissue perfusion is also important in patients with CLTI [5].

Many studies on this subject are observational and are heterogeneous in terms of study design, characteristics of the study sample, types of lesions and definitions of clinical outcomes. These aspects, combined with differences in revascularization techniques such as endovascular interventions and surgical bypass, complicate comparisons between studies [28,33]. This sharp dichotomy (direct/indirect flow) eventually diminishes the predictive value of the angiosome model as it does not take into account the possible microcirculation issues, typical of the small artery disease (SAD)/medial arterial calcification (MAC), the amount of collateral reserve and the clinical correlation with the type of wound (depth, magnitude, presence of osteomyelitis) [45].

Overall, although current evidence suggests that direct angiosome-guided revascularization may improve wound healing outcomes in selected patients with CLTI and DFU, many experts advocate for a pragmatic approach where restoring adequate perfusion to the ischemic area remains the primary therapeutic objective, regardless of whether revascularization is achieved through direct or indirect arterial pathways [15,28].

Most observational studies suggest that DR based on the angiosome concept may improve wound healing in CLTI patients [4,7,9,15,28]. However, evidence regarding limb salvage and long-term outcomes remains heterogeneous [7,9,19,46]. Despite the growing body of literature supporting angiosome-guided revascularization, several important limitations should be acknowledged. Most available studies are observational in nature and are subject to selection bias, as the choice between DR and IR is often influenced by anatomical feasibility rather than randomized allocation [7,9,15]. Additionally, there are variations between different studies, in patient populations, comorbidities, wound characteristics and clinical outcome definitions like wound healing and limb salvage [7,9,19]. Another variable consists of the many revascularization techniques, including endovascular and surgical approaches. All these differences make direct comparisons more complicated [15,28]. Moreover, many studies do not take into account the roles of collateral circulation and microvascular dysfunction, which may significantly influence tissue perfusion independently of angiosome targeting [16,17]. These limitations not only raise the need for cautious interpretation of current evidence but also support complementary frameworks like the woundosome concept, which incorporates functional perfusion [19,30].

Based on the current evidence, direct angiosome-guided revascularization should generally be preferred when the wound-related artery is technically accessible and can be treated safely [4,7,43]. However, this strategy should not be followed rigidly in all cases. In patients with complex anatomy, severe calcification, poor distal targets, or well-developed collateral circulation, indirect revascularization may still provide sufficient perfusion for wound healing, particularly when pedal arch continuity and functional perfusion are preserved [19,40,46]. In this regard, the angiosome and woundosome concepts should be viewed as complementary rather than competing approaches: the angiosome helps define the anatomical target, while the woundosome focuses on whether effective perfusion of the wound has actually been achieved.

### 3.4. Angiosome–Woundosome Integrated Perspective

While the angiosome model is still a cornerstone of vascular anatomy, modern clinical decision-making in the context of CLTI demands a more sophisticated, integrative approach. In many cases, wound healing depends not only on restoring flow to the source infra-popliteal artery of a specific angiosome, but also on the overall capacity of the infra-malleolar arterial network and the microcirculation to deliver effective perfusion to the wound bed [19].

From this perspective, the angiosome and woundosome concepts are not competing ideologies but rather compatible frameworks. The angiosome delivers the foundational anatomical map of vascular territories. In contrast, the woundosome shifts the focus toward the functional perfusion of the wound bed itself, accounting for the dynamic influences of collateral circulation, microvascular health, and the specific physiological conditions of the local tissue. By merging anatomical mapping with a functional assessment of tissue perfusion, clinicians may develop better revascularization strategies. In practical terms, the following steps can be considered [19,20,46]:

1. Wound localization: precise identification of the ulcer or tissue loss location helps define the affected angiosomal territory.
2. Angiosome mapping: the source artery supplying the wound-related angiosome should be identified, together with the anatomical feasibility of direct revascularization.
3. Assessment of collateral circulation: angiographic evaluation should include collateral pathways and potential cross-perfusion between adjacent angiosomes.
4. Technical feasibility of revascularization: lesion characteristics, vessel patency, distal runoff, and the likelihood of successful endovascular or surgical revascularization must be considered.
5. Functional perfusion assessment: when available, techniques such as pedal acceleration time (PAT), indocyanine green (ICG) fluorescence angiography, hyperspectral imaging, or other perfusion imaging modalities may help evaluate microcirculatory perfusion of the wound bed.
6. Target vessel selection: anatomical vascular mapping and functional perfusion findings should be integrated to identify the artery most likely to restore effective wound perfusion and support wound healing.

**Figure 4:**
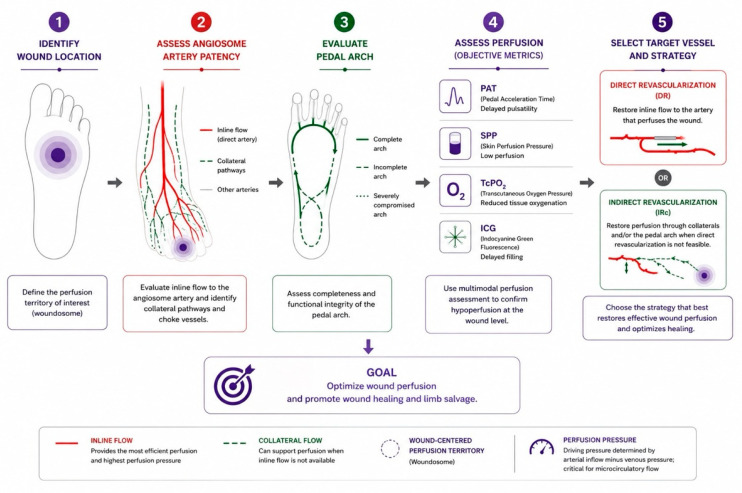
Practical clinical algorithm for wound-centered revascularization in chronic limb-threatening ischemia (CLTI). The schematic workflow illustrates a stepwise approach to revascularization planning based on wound localization, assessment of angiosome artery patency and collateral circulation, pedal arch evaluation, and multimodal perfusion assessment using pedal acceleration time (PAT), skin perfusion pressure (SPP), transcutaneous oxygen pressure (TcPO_2_), and indocyanine green (ICG) fluorescence imaging. Final target vessel selection is guided by restoration of effective wound perfusion and promotion of wound healing.

The practical clinical application of the woundosome concept in revascularization planning is illustrated in Figure 4.

This approach integrates both the angiosome and woundosome concepts, combining the anatomical model with functional perfusion considerations to improve outcomes in patients with CLTI. As imaging technologies and microcirculatory assessment tools continue to evolve, wound-oriented revascularization strategies could be developed to improve outcomes in patients with CLTI.

Currently, several classification systems are used for the management of CLTI cases. The Global Limb Anatomic Staging System (GLASS) is used to evaluate wound complexity and to plan the intervention, while the Wound, Ischemia, and foot Infection (WIfI) classification stratifies clinical severity and risk [11,17,34]. A major limitation of both systems is their inability to measure functional perfusion to the wound bed directly [17,18]. In particular, GLASS includes a pedal modifier for the infra-malleolar circulation, but this anatomical segment remains largely neglected and is not anatomically considered in the evaluation of the CLTI patients in major trials [17,47]. Including a more detailed description of the below-the-ankle circulation in major trials, as described by Karawada or, more appropriately, by Alexandrescu, remains difficult to achieve at the moment, although it could be very useful to understand the role of distal circulation in wound healing, especially because a high value of the MAC score has been demonstrated to be the most important and independent factor correlated with limb loss [38,45,48,49]. In this context, the woundosome concept, which covers anatomical factors, clinical aspects and effective tissue perfusion, may constitute a complementary framework [19]. By focusing on effective tissue perfusion rather than predefined anatomical territories alone, this approach could improve individualized treatment strategies in patients with CLTI.

A practical wound-centered revascularization workflow integrating both anatomical and functional perfusion assessment is illustrated in Table 4.

## 4. Discussion

### 4.1. Limitations

The angiosome concept has several limitations [31,50]. Natural variations in vascular anatomy, the frequent presence of multilevel arterial disease and the variable functional impact of collateral circulation can all reduce the relevance of an angiosome-guided strategy. Treatment selection also depends on the technical feasibility of the endovascular procedure [50,51]. The noted variability in clinical outcomes observed after angiosome-guided revascularization has consistently pointed out these limitations. Specifically, collateral circulation, macrovascular flow and microvascular tissue perfusion play a role in the healing process, independently of the angiosome that is targeted during revascularization. These aspects gave rise to the woundosome concept which highlights the functional perfusion of the wound bed rather than strict adherence to predefined anatomical vascular regions [16]. Its limitation is due to the actual lack of standardization of distal wound-related perfusion. Between many different methods, at the moment, the PAT appears to be the only reproducible and clinically validated way to assess distal perfusion, despite being operator dependent and still not included in any guideline [52].

### 4.2. Contemporary Revascularization Strategies and Functional Perfusion Assessment

Current revascularization strategies in chronic limb-threatening ischemia (CLTI) are increasingly focused on restoring effective tissue perfusion rather than relying only on anatomical target vessel selection. Although angiosome-guided revascularization remains clinically relevant, wound healing and limb salvage are influenced by several additional factors, including inline flow, collateral circulation, pedal arch integrity, perfusion pressure, and microvascular function.

The BEST-CLI trial further emphasized the importance of individualized revascularization planning based on anatomical complexity, conduit availability, patient comorbidities, and the long-term durability of perfusion restoration [53]. In this setting, the woundosome concept may offer a complementary functional perspective by integrating anatomical vascular territories with wound-centered perfusion assessment.

While direct revascularization should generally be preferred when technically feasible, indirect revascularization through collateral pathways or pedal arch recruitment may still provide adequate perfusion for wound healing in selected patients. In addition, studies comparing WIfI staging with angiosome-guided strategies suggest that wound severity, ischemic burden, and infection status may be more predictive of clinical outcomes than anatomical territorial matching alone [54].

Functional perfusion assessment using modalities such as skin perfusion pressure (SPP), transcutaneous oxygen pressure (TcPO_2_), pedal acceleration time (PAT), indocyanine green (ICG) fluorescence imaging, and perfusion imaging may further improve individualized target vessel selection and support wound healing within the woundosome framework.

### 4.3. Future Perspectives

Future research in CLTI is increasingly focusing on integrating anatomical and functional approaches to optimize revascularization strategies. The angiosome concept provides an important anatomical framework for understanding lower limb tissue perfusion; however, increasing evidence suggests vascular territories alone may not fully explain advanced peripheral arterial disease and diabetes patients’ complex perfusion patterns [19,30]. The new woundosome concept improves the angiosome model by taking into account the functional perfusion of the wound bed [30].

One of the most exciting future directions is represented by advanced perfusion imaging technologies, such as indocyanine green fluorescence angiography, hyperspectral imaging, perfusion angiography and laser Doppler flowmetry, which assess microvascular blood flow in real time. Their implementation into clinical practice would allow clinicians to identify the exact arterial source that restores perfusion in patients with CLTI, using both the anatomical angiosome model and the functional woundosome concept [15,30,37].

The next step would be to use this imaging for patient-specific hemodynamic modeling. Advances in artificial intelligence and computational vascular simulations may soon allow doctors to predict how perfusion will change after a revascularization procedure, which would be especially useful for complex cases with multilevel arterial disease or considerable collateral circulation [15].

Ultimately, a move toward more flexible, hybrid revascularization strategies is expected. This would be achieved by combining the anatomical aspects of angiosome-guided revascularization with the functional perspective of the woundosome concept. In selected patients, especially when direct arterial access is problematic, IR through collaterals may lead to outcomes that are comparable to angiosome-targeted DR [19,28].

Future prospective studies and randomized clinical trials are required to compare the angiosome-guided and wound-targeted revascularization strategies in patients with CLTI, in terms of objective perfusion assessment and standardized clinical outcomes, such as wound healing, limb salvage and patient-reported outcomes [15,19]. Used together, angiosome anatomy and woundosome functional perfusion concepts could result in a more personalized approach to limb salvage. Revascularization strategies that are tailored to individual vascular anatomy, collateral circulation and microcirculation may represent a future paradigm for CLTI management.

The woundosome concept appears to be supported by emerging clinical observations, but its integration into practice is still limited by the absence of high-quality prospective evidence. There is a need for comparative studies that evaluate woundosome-oriented, conventional angiosome-based and non-targeted revascularization in patients with DFU and CLTI. These studies should include patient cohorts that are comparable and should assess clinical outcomes, wound healing, limb salvage, feasibility of the procedure and cost-effectiveness. This type of comparative data could be used to determine the clinical value and practical applicability of perfusion strategies based on the woundosome concept. This paradigm currently lacks validation through large-scale, reproducible clinical studies. New therapeutic frameworks in vascular medicine can only be adopted based on statistical confirmation across comparable study designs, including both univariate and multivariate analyses.

## 5. Conclusions

This review supports a shift from purely anatomical revascularization strategies toward a functional, wound-centered approach. Integrating angiosome and woundosome concepts may provide a more precise and clinically actionable framework for optimizing revascularization in CLTI.

The angiosome concept remains a good anatomical framework that can be used to guide revascularization strategies in patients with CLTI. However, increasingly more evidence has shown that anatomical vascular maps alone may not reflect the complexity of tissue perfusion and wound healing.

The woundosome concept is a complementary perspective based on the functional perfusion of the wound bed that takes into account direct arterial inflow, collateral circulation and microvascular integrity. It allows for a more personalized selection of the target vessel and may better reflect the real perfusion patterns of the patient.

This individualized strategy does not seek to replace the angiosome model but rather to enhance it. By adopting a combined angiosome–woundosome framework, clinicians can make more informed clinical decisions. This is especially useful in complex cases where DR cannot be done or where collateral circulation is significant.

Moving forward, future studies should aim to validate wound-oriented perfusion models using objective perfusion imaging and standardized clinical parameters such as wound healing, limb salvage, and patient-centered outcomes.

This integrated approach represents progress toward more precise, functionally guided and patient-specific personalized revascularization strategies in CLTI.

## Figures and Tables

**Table 1:** Angiosome territories of the foot [2,3,20].

Source Artery	Angiosome	Main Territory	Clinical Relevance
Posterior tibial artery	Medial plantar	Medial plantar instep	Plantar midfoot ulcers
Posterior tibial artery	Lateral plantar	Plantar forefoot and lateral plantar foot	Plantar forefoot ulcers
Posterior tibial artery	Calcaneal	Plantar heel	Heel ulcers
Anterior tibial artery (dorsalis pedis)	Dorsal foot angiosome	Dorsum of the foot and toes	Dorsal ulcers
Peroneal artery	Anterolateral ankle	Anterior-lateral ankle	Ankle wounds
Peroneal artery	Lateral calcaneal	Lateral heel	Lateral heel ulcers

**Table 2:** Key studies supporting the woundosome concept in CLTI, published in 2021–2026 [5,16,19,20,36,38,39,40].

Study	Year	Study Type	Population/Model	Main Concept	Key Findings
Tarricone et al. [5]	2026	Systematic review and meta-analysis	985 patients with CLTI; analysis of 9 primary studies	DR vs. IR	The selection of a revascularization method appears to have little effect on wound healing time, as clinical outcomes are influenced by multiple factors, including post-procedure care and the optimization of the patient’s physiological status.
Moysidis et al. [19]	2025	Narrative review	Patients with CLTI and DFU; analysis of 14 primary studies	Angiosome vs. woundosome concept	Introduces the woundosome concept as a functional extension of the angiosome model, emphasizing perfusion of the wound bed. According to the woundosome concept, the results of IR are comparable to those achieved through angiosome-based DR methods, provided that the wound area has appropriate collateral circulation; The woundosome paradigm provides increased adaptability for treating patients with intricate vascular anatomies; its practical application depends on the use of instruments for measuring tissue perfusion.
Miyake et al. [36]	2024	Single-center retrospective cohort study	117 limbs (108 patients) with CLTI and foot wounds with bypass surgery (popliteal, crural, or pedal arteries)	Assessing how pedal circulation status, visualized through angiography, and WIfI classification influence graft patency and wound healing.	The key predictors of delayed or unsuccessful wound healing are an increased WIfI stage and non-visualized wound perfusion; The distribution of blood supply to the wound site is more significant for successful healing than the overall perfusion to the foot; Visualized arterial flow to the wound site is linked to improved graft patency; These outcomes validate the effectiveness of bypass strategies guided by the woundosome concept for treating complex foot ulcers; Anatomical staging systems should include pedal perfusion status for better clinical decision-making and patient recovery.
Serizawa et al. [40]	2024	Prospective multicenter observational study	29 lower limbs evaluated in 27 patients with CLTI.	Measuring SPP after DR vs. IR to determine if treatment efficacy depends on the angiosome concept	Distal bypass improves foot SPP regardless of the revascularized angiosome; Wound healing had higher success rates for IR, although the IR group was smaller.
Sahebalzamani et al. [16]	2024	Cross-sectional study	84 patients (89 lower limbs evaluated) with DFU	Analysis of the validity and clinical applicability of the angiosome concept for DFU	The angiosome concept provides inconsistent support for explaining ulcer location; Collateral circulation should be taken into consideration.
Tange et al. [20]	2023	Prospective cohort study	52 patients (54 limbs) with lower extremity arterial disease	Used indocyanine green near-infrared fluorescence imaging to measure microvascular flow changes after DR and IR	DR and IR both result in significant perfusion improvement, with no statistical difference between strategies; Findings suggest that collaterals have a role in foot oxygenation.
Alexandrescu et al. [38]	2022	Retrospective analysis	336 ischemic feet (221 in diabetic patients) with Rutherford 5 CLTI	The introduction and validation of a 4-grade morphological classification (grades A-D) for BTA arterial disease in patients with CLTI	BTA morphological grading (A-D) is a reliable predictor for limb salvage; The angiosome model is effective for early-stage disease (A-B), but it reaches its clinical limit in the case of “desert foot” (D), where anatomical landmarks disappear and endovascular therapy is limited; Successful revascularization requires a global evaluation of the integrity of angiosomal source artery, foot arches, and collateral vessels network, the “physiologic angiosome”.
Kim et al. [39]	2021	Systematic review and meta-analysis	4252 limbs (2231 DR; 1647 IR; 270 IR though collateral vessels) in patients with critical limb ischemia	Comparison of DR, IR, and IRc in treating lower extremity wounds with the angiosome concept.	DR and IRc result in significantly higher wound healing and limb salvage vs. IR; IRc has comparable wound healing outcomes when DR cannot be performed.

BTA: below-the-ankle; CLTI: chronic limb-threatening ischemia; DFU: diabetic foot ulcer; DR: direct revascularization; IR: indirect revascularization; IRc: indirect revascularization through collateral vessels; SPP: skin perfusion pressure; WIfI: Wound, Ischemia, and Foot Infection.

**Table 3:** Selected studies evaluating angiosome-guided revascularization in patients with chronic limb-threatening ischemia (CLTI), published between 2021–2026 [5,14,19,21,30].

Study	Year	Study Design	Population/Model	Revascularization Type	Main Outcomes	Key Findings
Tarricone et al. [5]	2026	Systematic review and meta-analysis	985 CLTI patients from 9 clinical studies	Mixed (EVT and surgical bypass)	AFS, wound healing (6 months), and survival for DR vs. IR compared across studies	DR is associated with improved AFS, binary wound healing, and overall survival compared to IR.
Moysidis et al. [19]	2025	Narrative review	Patients with CLTI and DFU	EVT and surgical (bypass)	Comparison of wound healing and limb salvage between the angiosome approach and the woundosome strategy	Highlights limitations of the angiosome model and introduces the woundosome concept.
Meloni & Vas [14]	2025	Review	Patients with ischemic DFU	Surgical bypass, EVT, cell therapy	Relation between three PAD patterns, healing rates and the major amputation risk	BTA disease and SAD have the highest amputation risk; SAD requires autologous cell therapy; Revascularization should prioritize the wound-related artery.
Popitiu et al. [30]	2024	Retrospective observational study	51 patients (51 limbs) with Rutherford 5–6 CLTI	EVT-DCB vs. plain balloon	Ulcer healing, limb salvage, and AFS in DR vs. IR and DCB vs. plain balloon	DR significantly improves limb salvage and healing; Better clinical outcomes were achieved with DCB.
Hou et al. [21]	2022	Retrospective study	112 legs in 111 patients with DFU and PAD	EVT-percutaneous transluminal angioplasty/stent	Ulcer healing rate, mean time to healing, survival rate, and major amputation-free survival (AFS) within a 1-year follow-up period.	Angiosome-guided revascularization had better wound healing outcomes and survival than IR.

AFS: amputation-free survival; BTA: below-the-ankle; CLTI: chronic limb-threatening ischemia; DCB: drug-coated balloon; DFU: diabetic foot ulcer; DR: direct revascularization; EVT: endovascular therapy; IR: indirect revascularization; PAD: peripheral arterial disease; SAD: small artery disease.

**Table 4:** Complementary roles of the angiosome and woundosome concepts in chronic limb-threatening ischemia (CLTI) revascularization planning. The table summarizes the key anatomical and functional features of the angiosome and woundosome concepts and illustrates how these approaches may be integrated to support personalized wound-centered revascularization strategies.

Clinical Aspect	Angiosome Concept	Woundosome Concept	Integrated Clinical Implication
Main focus	Anatomical vascular territory	Functional wound perfusion	Combined anatomical-functional assessment
Target selection	Source artery to wound angiosome	Effective perfusion to wound bed	Personalized revascularization planning
Collateral circulation	Secondary role	Central role	Assessment of collateral reserve
Pedal arch	Anatomical continuity	Functional perfusion support	Evaluation of distal perfusion
Imaging	Angiography	SPP, TcPO2, PAT, ICG	Multimodal perfusion assessment
Clinical objective	Direct arterial reperfusion	Restoration of wound perfusion	Optimization of wound healing and limb salvage

## Data Availability

No new data were created or analyzed in this study.

## Abbreviations

The following abbreviations are used in this manuscript:

AFS	amputation-free survival
ATA	anterior tibial artery
BTA	below-the-ankle
CLTI	chronic limb-threatening ischemia
DCB	drug-coated balloon
DFU	diabetic foot ulcer
DR	direct revascularization
EVT	endovascular therapy
GLASS	Global Limb Anatomic Staging System
IR	indirect revascularization
IRc	indirect revascularization through collateral vessels
MAC	medial arterial calcification
PAD	peripheral arterial disease
PAT	pedal acceleration time
PTA	posterior tibial artery
SAD	small artery disease
SPP	skin perfusion pressure
WIfI	Wound, Ischemia, and Foot Infection
